# The Connection Between Oxidative Stress, Mitochondrial Dysfunction, Iron Metabolism and Microglia in Multiple Sclerosis: A Narrative Review

**DOI:** 10.3390/neurosci6010023

**Published:** 2025-03-04

**Authors:** Simonida Delic, Svetlana Miletic Drakulic, Milos Stepovic, Jovana Milosavljevic, Marija Kovacevic Dimitrijevic, Kristijan Jovanovic, Ivona Marinkovic, Melanija Tepavcevic, Nikoleta Janicijevic, Aleksandra Mitrovic, Danica Igrutinovic, Maja Vulovic

**Affiliations:** 1 Department of Anatomy, Faculty of Medical Sciences Kragujevac, University of Kragujevac, 34000 Kragujevac, Serbia; stepovicmilos@yahoo.com (M.S.); jowana.ilic@yahoo.com (J.M.); marijakovacevic.mk@gmail.com (M.K.D.); kralj100@yahoo.com (K.J.); ivbankovic1@gmail.com (I.M.); melanijatepavcevic@yahoo.com (M.T.); maja@fmn.kg.ac.rs (M.V.); 2 Department of Neurology, Faculty of Medical Sciences Kragujevac, University of Kragujevac, 34000 Kragujevac, Serbia; mileticdrakulic@gmail.com; 3 Department of Hygiene and Ecology, Faculty of Medical Sciences Kragujevac, University of Kragujevac, 34000 Kragujevac, Serbia; nikoleta.janicijevic@gmail.com; 4 Department of Gynecology and Obstetrics, Faculty of Medical Sciences Kragujevac, University of Kragujevac, 34000 Kragujevac, Serbia; mitrovicaleksandra573@gmail.com; 5 Department of Biochemistry, University Clinical Centre Kragujevac, 34000 Kragujevac, Serbia; danica7pavlovic@gmail.com

**Keywords:** multiple sclerosis, microglia, oxidative stress, mitochondrial dysfunction, iron deposition

## Abstract

In recent years, in the pathogenesis of multiple sclerosis, emphasis has been placed on mitochondrial processes that influence the onset of the disease. Oxidative stress would be one of the consequences of mitochondrial dysfunction, and its impact on brain tissue is well described. Microglia, as a brain macrophage, have an important function in removing unwanted metabolites, as well as iron, which is an amplifier of oxidative stress. There are novelties in terms of the connection between these processes, which have redirected research more towards the process of neurodegeneration itself, so that the emphasis is no longer on neuroinflammation, which would initiate the pathological process itself and still exist in the vicinity of lesions with reduced intensity. The aim of this review is to summarize the current knowledge from the literature regarding oxidative stress, mitochondrial dysfunction and iron metabolism and how microglia are involved in these processes in multiple sclerosis.

## 1. Introduction

Multiple sclerosis is an autoimmune, neuroinflammatory, neurodegenerative and demyelinating disease of the central nervous system (CNS). The disease is multifactorial, with genetic and environmental factors at the core [1]. Neuroinflammation and neurodegeneration coexist from the beginning, except that neuroinflammation dominates in the relapsing-remitting form, while neurodegeneration dominates in the primary-progressive and secondary-progressive forms [2]. In diagnosis, it is important to meet the criteria for spatial and temporal dissemination of lesions, which belong to the McDonald criteria [3]. Given that the degeneration is an unstoppable process, complete curing is not currently possible, only disease control [3,4].

Nervous tissue does not have the ability to regenerate, but neuroplasticity and remyelination are compensatory processes, an attempt to restore damaged myelin. Myelin is produced by oligodendrocytes, glial cells. The disease progresses more slowly if remyelination in the lesions is intense. Remyelination takes approximately 6 months [5]. The remyelination process is usually partial, with astrocytes forming a scar [2]. The basic processes in multiple sclerosis are neuroinflammation and neurodegeneration. Immune system cells, astrocytes and microglia participate in neuroinflammation [5]. Neurodegeneration is a process that is not immediately obvious because it only begins to manifest as disease progression with a longer duration of the disease. In this process, where axons and neuron bodies are damaged, neurofilament light chain (Nfl) is released, which is used as a biomarker for monitoring the outcome because it is associated with relapses and disease progression. One of the important processes is calcium channel dysfunction. Increased sodium concentration in lesions has also been found [5].

The brain is highly susceptible to oxidative stress due to its high oxygen consumption, high lipid content in the myelin sheath, and low levels of antioxidant enzymes compared to the liver. It also contains high levels of free iron and ascorbate. Physiological concentrations of free radicals are signaling molecules in cellular processes. They are generated by mitochondria during the process of cellular respiration in the mitochondrial respiratory chain. Free oxygen radicals are superoxide anion, hydroxyl radical and hydrogen peroxide, but there are also nitrogen radical derivatives. In antioxidant defense, the most important enzymes are superoxide dismutase, glutathione peroxidase, catalase and heme oxygenase. Compounds that bind free radicals are uric acid, glutathione, ascorbates, α tocopherol, and carotenoids [3]. In addition to free radicals, the neurotransmitter glutamate also contributes to myelin damage [2].

## 2. A New Concept of Multiple Sclerosis: A Smurring Disease

Lublin redefined the terms related to disease progression. Multiple sclerosis activity includes the presence of relapses, imaging features (contrast binding T1 lesions or new or enlarging T2 lesions), and the presence of relapse-independent progression that occurs in secondary progressive and primary progressive forms of multiple sclerosis. It is necessary to observe the development of the disease over a period of time, such as a year. Deterioration includes the presence of either relapses or relapse-independent progression [6].

The understanding of the basic disease process has changed and is now viewed as latent, smoldering activity independent of relapses and focal activities on magnetic resonance imaging, which are no longer good predictors of multiple sclerosis progression. The accumulation of disability occurs from the onset of the disease. The goal where achieving the status of no evidence of disease activity (NEDA) is no longer adequate. Clinical deterioration occurs despite the absence of inflammation, relapse, new contrast-enhancing T1 or new or enlarging T2 lesions [7].

In a study involving approximately 35,000 patients with follow up of up to 15 years, it was examined how much relapses contribute to worsening of the disease and the accumulation of new disabilities. According to the mechanism of disability, the deterioration was divided into relapse-associated (RAW)—representing deterioration caused by relapse; and progression independent of relapse activity (PIRA)—representing deterioration independent of relapse activity [8].

Smoldering multiple sclerosis is actually a form of PIRA characterized by progression without relapse. The emphasis is on a primarily smoldering latent process, while inflammation is secondary to it. Suppression of relapses does not prevent the accumulation of disability. There is a new understanding that the disease begins in the CNS where neurons are primarily damaged and myelin fragments are triggers for the activation of the immune response. In primary progressive and secondary progressive multiple sclerosis, similar lesions have been found pathoanatomically, with a similar time of progression and a similar degree of disability. The number of relapses decreases with age. The risk of the secondary progressive form decreases as the person ages. Age-related neurodegenerative processes accelerate the progression of disability. The consequence is a loss of brain tissue volume that may be a predictor of the development of multiple sclerosis. The question arises whether axons in the CNS can regenerate as peripheral neurons. Demyelinated axons are less resistant to environmental excitotoxicity, which, together with the failure of remyelination, contributes to the occurrence of the disease [7].

Microglia and macrophages have been found in multiple sclerosis lesions. They are thought to be responsible for neuronal loss. Slow-growing lesions are a low-grade inflammatory substrate where iron-laden phagocytes are surrounded by proliferating oligodendrocytes. Slow-growing lesions do not shrink, but rather expand because the ring of microglia at the edges destroys the surrounding parenchyma and, due to their sensitivity, are less likely to remyelinate. These lesions indicate a much more severe clinical picture and are the cause of cognitive decline in patients. Positron emission tomography (PET) scans detect these lesions well. Low levels of inflammatory activity were found in microglia in normal-appearing gray and white matter, which may precede lesion formation. In the secondary progressive form, these changes were associated with the accumulation of disability and brain atrophy [7].

According to the histological classification, there are four types of active lesions. In the third and fourth types of lesions, damage to oligodendrocytes is encountered. Microglia cells are present in all four types of lesions. Cortical lesions are observed only in the secondary progressive form and primary progressive forms. Active lesions with remyelination are called “shadow plaques”. Inactive lesions are called smoldering, slowly expanding, and in them the center is hypocellular while there is a ring of microglia around them. In them, axonal loss is noticeable [9].

Acute axonal damage is most intense early in the course of the disease and depends on the activity of the lesion. The potential for remyelination is limited, depending on the age and duration of the disease. In the progressive form, there is a chronic active lesion, microglia activation, and infiltrate in the meningeal membranes [10]. In the progressive form of the disease, there is a decrease in the number of lesions and increased atrophy of structures without focal inflammation. The process is compartmentalized in the CNS where there are chronic active, expanding lesions surrounded by a ring of microglia. In addition, there are cortical and deep gray matter lesions, diffuse microglial activation seen on PET scans, and lymphatic aggregates of the meningeal sheaths. In addition to proinflammatory cytokines, microglia can also release anti-inflammatory cytokines and participate in remyelination by mobilizing oligodendrocyte precursors [11].

The lesion itself contains Th1 lymphocytes, Th2 lymphocytes, Th17 lymphocytes, Treg lymphocytes, B cells, and myeloid suppressor cells. Apoptotic oligodendrocytes with focal clusters of microglia can be found in new lesions. Microglia and macrophages release proinflammatory mediators and free radicals and also contain myeloperoxidase, which produces hypochlorous acid. Lipid and DNA oxidation occurs in the lesion itself [12].

There is oxidative damage in the surrounding lesion tissue. In chronic lesions in multiple sclerosis, significantly fewer oligodendrocyte progenitors are observed than in normal-appearing tissue. Chronic active lesions have a clear demarcation with macrophages at the edges of the plaques, while the center of the lesion is less active. Smoldering plaques contain linear aggregates of microglia around short segments of disrupted myelin. Oligodendrocytes are absent in the center of the lesion. Chronic inactive plaques are hypocellular without oligodendrocytes and axons, with astrogliosis, and without immune cells [13].

## 3. Biological Markers of Oxidative Stress

Free radicals can cause lipid peroxidation of phospholipids of cell membranes. A chain reaction occurs that spreads to lipid-rich regions. In the presence of reduced metals and ascorbate, malonaldehyde, acrolein 4-hydroxy-2-nonenal and 4-hydroxy-2-hexenal are formed, which have a long half-life and can diffuse to distant sites. They interact with the amino acids cysteine, histidine and lysine, which make up one third of the myelin sheath [3] (Table 1).

Oxidative stress and mitochondrial dysfunction lead to the loss of adenosine triphosphate (ATP), the energy responsible for key processes. The formation of free radicals from cell membrane lipids, the accumulation of iron in the nuclei of the gray matter, and in the lesion ring formed by phagocytes are the consequences of oxidative stress [5].

Reactive oxygen and nitrogen radicals are superoxide anion, hydroxyl radical, peroxyradicals, nitrogen monoxide, hydrogen peroxide, singlet oxygen and peroxynitrate. Low levels of free radicals act as secondary messengers for signal transduction in cells. The protective mechanism is activated by nuclear factor-like transcription factor 2 (Nrf2) which further leads to the expression of antioxidant proteins, enzymes, transporters such as glutathione synthetase, thioredoxin enzyme system, heme oxygenase, and NADPH quinone oxidoreductase [12].

The sulfur atom in cysteine, as a part of GSH, destroys free radicals. Quinones and free heme lead to the formation of free radicals. Nrf2 binds Kelch ECH-associating protein (Keap1) in the cytosol, while oxidative stress causes conformational changes that prevent this binding. Nrf2 then translocates to the nucleus, where it activates the expression of the antioxidant response element (ARE) [12].

## 4. Mitochondrial Dysfunction

The brain has a lower density of mitochondria but uses 10 times more oxygen than other tissues. Oxidative phosphorylation occurs on the ridges of the inner membrane. The brain has a reduced antioxidant defense compared to other tissues. Nerve cells are metabolically highly active, postmitotic, and irreplaceable. Mitochondria of neurons have a larger mass. In addition to ATP production, their role is in the formation of iron–sulfur clusters, calcium storage, cell death pathways, and free radical signaling pathways [23].

The brain mainly uses glucose to create ATP. Gray and white matter have different energy requirements, because gray matter has synapses, while white matter and glia are pathways. Astrocytes are metabolic support for neurons and glycolysis mainly takes place in them to obtain energy. Mitochondria produce 16 times more ATP than in the process of glycolysis. ATP is also necessary for the development of axons, dendrites, axon regeneration, synaptic transmission and plasticity. Astrocytes release lactate during glycolysis, which is taken up by neurons for oxidative phosphorylation. Astrocytes also metabolize fatty acids in the cerebellum due to the enzymes acyl coenzyme A dehydrogenase and carnitine palmitoyl transferase 1a, and they also possess peroxisomal proteins (catalase) [25].

Astrocytes are metabolically active cells that can donate their mitochondria to damaged neurons. If astrocyte mitochondria are damaged, energy production decreases and the blood–brain barrier is disrupted [26]. In astrocytes, there is a high production of free radicals and a reduced process of cellular respiration, while in neurons, there is a high level of mitochondrial respiration at the supercomplexes and a reduced production of free radicals [23,25].

Mitochondrial DNA is more susceptible to mutations because it lacks histones, has limited repair, and is constantly exposed to oxidative stress. Lactate is a product of anaerobic metabolism and was the first biomarker used for mitochondrial dysfunction [22]. In the process of oxidative phosphorylation, free radicals are generated due to electron transfer in the transport chain, as well as during the tricarboxylic acid cycle and fatty acid metabolism. Increased glucose metabolism leads to the accumulation of lactate. In active lesions, mitochondrial activity is higher, while in inactive lesions, mitochondrial mass and activity of complex 4 of the respiratory chain are increased [21].

In cases of oxidative phosphorylation disorders, energy production shifts to anaerobic glycolysis, where lactate is produced, or β-oxidation of fatty acids is activated. During oxidative phosphorylation, if electrons leak from the electron transport chain, they react with oxygen and superoxide anion is produced. Research has focused on anaerobic glycolysis and the tricarboxylic acid cycle, which are alternative pathways for obtaining energy, iron deposition, which is detected by iron-sensitive contrast on magnetic resonance imaging, or on neuroinflammatory markers, which are detected by PET imaging or fluid-sensitive magnetic resonance imaging (MRI) [27]. Mitophagy (autophagy of mitochondria) is also disrupted, and mitochondria are damaged and unrecognizable [23].

There is a difference between nonsynaptic and synaptic mitochondria. Complex 1 is actually a regulator of degenerative processes characterized by synaptic dysfunction. In synaptic mitochondria, lower levels of pyruvate dehydrogenase were found, as well as reduced expression of complexes 1, 2, and 4. Synaptic mitochondria had an increased number of age-related deletions and reduced levels of antioxidant enzymes, indicating reduced defense against free radicals [25].

Ceramides are mediators of mitochondrial dysfunction because they induce the formation of free radicals and alter mitochondrial membrane permeability, leading to neuronal death. In rats with experimental autoimmune encephalomyelitis, mitochondria are elongated and the activity of complexes 1, 3, 4 is reduced [14].

Reduced glycolysis, oxidative phosphorylation activity, and reduced mitochondrial membrane potential were observed. Increased extracellular acidification and increased oxygen utilization were observed during relapse. Teriflunomide reduces the activity of these processes and complex 3 of the respiratory chain. In the primary progressive form, apoptosis of immune cells is reduced. In the relapsing form, an increase in the activity of glucose transporters is registered, which leads to an increase in lactate. Increased oxidative phosphorylation activity leads to the development of the Th17 line of immune cells. Lack of the transcription factor BATF is associated with resistance to multiple sclerosis. Deletion of the Nur77 gene promotes the development of a proinflammatory phenotype of immune cells, which leads to exacerbation of multiple sclerosis [21].

Lactate is also elevated in the cerebrospinal fluid of patients with multiple sclerosis. CD8 T lymphocytes have mitochondria of greater mass and membrane potential in the relapsing form with increased expression of the glucose transporter. In the secondary progressive form, periventricular damage to B lymphocyte mitochondria has been detected. Inhibition of mitochondrial respiration reduces B cell activation. In patients with multiple sclerosis, glycolysis is a more active process than in healthy individuals. In patients who did not respond to treatment with interferon beta, mitochondrial dysfunction is present. In those who responded, there was a slight decrease in the level of free radicals and improvement in mitochondrial dysfunction. In addition to free radicals, deterioration in the lesion is caused by mitochondrial dysfunction and iron release. It has been found that fibrin induces oxidative stress. In inflammatory-type macrophages of patients, the expression of glucose transporters in the lesion itself is increased. Inhibition of glycolysis by dimethyl fumarate reduces antigen presentation and mediators. In an experiment, a compound produced by the helminth Fasciola hepatica prevented the progression of multiple sclerosis by reducing glycolytic activity in macrophages. Lactate leads to a decrease in oxidative phosphorylation activity and free radical generation [21].

Free radicals are mainly produced at complexes 1 and 3. Even acute exposure to free radicals reduces energy production, because it inactivates iron–sulfur clusters. It has been established that an increase in intracellular calcium released from mitochondria causes neurodegeneration because it changes the membrane potential of mitochondria and thus increases the production of free radicals [23].

Mitochondrial dysfunction creates virtual hypoxia, via a defect in complex 4 (cytochrome C oxidase), which consequently results in disruption of ion homeostasis and neuronal death. Biotin deficiency has been observed in multiple sclerosis. The use of oxygen is still being considered in therapy [7].

It has been found that TNF-α affects oxidative phosphorylation via calcium levels. The presence of the Na^+^/K^+^ pump is increased, which increases the concentration of Na+ in neurons, the Na^+^/Ca^2+^ exchanger is activated, and calcium increases in neurons, which leads to apoptosis. The heat shock protein mtHSP70 has been cited as a marker of mitochondrial stress [2].

Previous studies emphasized the importance of studying mitochondrial dysfunction, which is a trigger for the apoptosis process. Therapeutic options for mitochondrial D^NA^ repair are being considered. Mitochondrial dysfunction also occurs in CNS glial cells, not just neurons. Another process associated with multiple sclerosis is increased mitophagy activity. Antioxidants such as alpha lipoic acid should be tried in therapy [28].

## 5. The Role of Microglia and the Influence of Iron in the Pathogenesis of Multiple Sclerosis

Microglia is a macrophage that originates from the mesenchyme, migrating from the yolk sac during embryonic development. The two phenotypes of microglia are M1 proinflammatory, neurotoxic and M2 anti-inflammatory, neuroprotective. The roles of microglia are self-renewal without precursors from the periphery, process control and pathogen removal, antigen presentation, synapse formation, myelin phagocytosis and release of growth factors for recovery in remyelination, participation in iron recycling, and enabling the survival of oligodendrocyte precursors by releasing BDNF (brain-derived neurotrophic factor), ILGF1 (insulin-like growth factor 1), activin A, semaphorin 3F and transglutaminase. Neurophilin, which it also releases, stimulates the formation of myelin [29,30].

Neurotoxic astrocytes destroy oligodendrocytes and neurocytes in the lesion. The M1 phenotype disrupts the differentiation of oligodendrocyte precursors into mature cells, while the M2 phenotype produces regenerative factors such as triggering receptor expressed on myeloid cells (TREM2) which affects the density of oligodendrocytes in the area where they are created [31,32].

Bruton’s tyrosine kinase inhibitors reduce the release of proinflammatory mediators by microglia while promoting remyelination. Examples include ibrutinib, tolebrutinib, which is still in the research phase and has been found to reduce TNFα levels, and ebrutinib, which promotes the development of the M2 phenotype [33].

Microglia direct the development of neurons during the embryonic stage. Microglia have four states: nonspecific, homeostatic, proinflammatory and anti-inflammatory. The proinflammatory state expresses ferritin, an iron storage protein, as a marker. Microglia express transmembrane protein 119(TMEM119) and the purinergic receptor (P2Y12). Even in normal-appearing tissue, activated microglia with increased expression of genes for lipid metabolism have been registered. Bone morphogenetic protein 4(BMP4) is expressed more in regions of remyelination. In microglia, translocator protein (TSPO), which is located on the outer membrane of mitochondria, is also expressed more in increased amounts, even in the surrounding normal-appearing tissue, most often in the secondary progressive form and in the clinically isolated syndrome. Natalizumab and glatiramer acetate have successfully reduced the binding of this molecule on PET imaging. TSPO correlates with EDSS score in progressive forms, T2 lesion enlargement in relapsing forms, and brain atrophy in secondary progressive forms. The problem is that patients are exposed to radiation and TSPO can be expressed in both macrophages and astrocytes [9].

Extracellular vesicles of microglia origin have been observed in tears and are thought to reflect levels of extracellular vesicles in CSF. CD163 is a receptor that binds the hemoglobin–haptoglobin complex and is elevated in both microglia and macrophages. Interferon beta reduces Major histocompatibility complex (MHC) class II expression on microglia but increases proinflammatory mediators. Glatiramer acetate, dimethyl fumarate, and teriflunomide (indirect effect) also had an inhibitory effect on microglia. Fingolimod has the greatest effect because it binds to S1P (sphingosine 1 phosphate receptor) on microglia. Natalizumab indirectly stimulates the release of neurotrophic factors. Minocycline is an antibiotic that has shown a beneficial effect on microglia in preclinical studies [9].

In neuroinflammation and neurodegeneration, there is constant activation of microglia. Mature oligodendrocytes (expressing myelin basic protein (MBP) and myelin oligodendrocyte glycoprotein (MOG)) bind to the remnant of myelin and thus the process of remyelination begins. Microglia also recruit oligodendrocyte progenitors to the lesion site using semaphorin 3F (Sema 3F). Interferon regulatory factor 8 (IRF8) and colony stimulating factor receptor 1 (CSF-R1) are essential for microglia development. CSF-R1 is overexpressed in the progressive form in areas of demyelination. Myelin debris inhibits the differentiation of oligodendrocyte progenitors. Astrocytes also participate in the removal of myelin debris because they stimulate oligodendrocyte progenitors with the help of leukemia inhibitory factor (LIF). In addition, microglia activate astrocytes via interleukin 1 β (IL1-β). Microglia can also phagocytize T lymphocytes, in addition to myelin and damaged neurons. Microglia are present in a ring at the edges of a chronic active lesion filled with iron in the progressive form after a 10-year duration. The M1 phenotype is present in the early phase of the lesion, while in the late phase the M2 phenotype of microglia dominates, which stimulates the differentiation of oligodendrocyte progenitors and releases growth factors (brain-derived neurotrophic factor (BDNF), neural growth factor (NGF), glial-derived neurotrophic factor (GDNF), neurophilin, insulin-like growth factor 1 (IGF-1) and colony stimulating factor 1 (CSF1)). According to their appearance, there are four types of microglia: branched, amoeboid, phagocytic and dystrophic. The M2 phenotype becomes dystrophic over time with deformed mitochondria and indicates the presence of inflammation [34]. Microglia cause persistent inflammation that later occurs without the presence of lymphocytes [28]. One study demonstrated that as neuroinflammation becomes chronic, microglia express less Pcr and ATP, lower levels of antioxidants, and a reduced nucleotide-binding oligomerization domain (NOD) and leucine-rich repeat (LRR)-containing protein (NLRP3) inflammasome response [35].

There is a dysfunction of synapses in which astrocytes and microglia play the main role, and neuropeptides as signaling molecules that participate in the work of synapses can be neuroprotective, but also neurotoxic. Neuroprotective effects were shown in experiments by vasoactive intestinal peptide (VIP), pituitary adenylate cyclase activating peptide (PACAP), calcitonin gene-related peptide (CGRP), and substance P and neuropeptide Y(NPY), while endothelins showed pathogenic effects [36].

In an experiment, rats were exposed to cerebrospinal fluid from patients with the progressive form, which led to a disruption in energy production.

Microglia create free radicals more in the presence of iron, as do oligodendrocytes in which iron accumulates. Iron also accumulates extracellularly after the death of oligodendrocytes, so microglia take them over and become degenerated and dystrophic. These changes are more common in the secondary progressive form. Many regions accumulate iron that does not participate in oxidative stress. Therefore, imaging is performed with sensitivity, such as iron-sensitive methods [14].

In the Fenton reaction, iron reacts with hydrogen peroxide, promoting the formation of free radicals. This disrupts mitochondrial function, releases free radicals, and affects synapses. Lipid peroxidation also exacerbates neuroinflammation and stimulates the release of iron from binding proteins. The iron is then taken up by microglia [23,37,38].

Iron levels are considered to be a generator and amplifier of oxidative stress. Iron-binding proteins (such as heme) or iron–sulfur clusters are sensitive to oxidative stress. De novo synthesis of iron–sulfur clusters occurs in mitochondria. Excess iron accumulates mainly in mitochondria, which causes oxidative stress. An important role in iron metabolism is played by the thioredoxin and glutaredoxin protein systems, which participate in the transfer and maturation of iron–sulfur clusters. Iron–sulfur binding complexes are sensors of iron status in the cell and are susceptible to oxidative degradation. Their levels are regulated by the iron regulatory protein–iron responsive element (IRP-IRE) system of iron regulatory molecules [39].

Recent articles have shown that inhibition of iron accumulation in microglia prevents neuroinflammation [40,41,42]. Ferroptosis (cell death caused by lipid peroxidation under the influence of iron) and chronic inflammation mediated by microglia can lead to neurodegeneration as a consequence. During ferroptosis, mitochondria shrink, ridges are lost, mitochondrial membrane density increases and outer mitochondrial membrane rupture occurs. Microglia remove pathological particles and phagocytize damaged tissue. In multiple sclerosis, iron accumulates in the microglia of chronic active lesions. The M1 type of microglia has been found at the margins of the lesion, which participates in the progression of the lesion. The M1 phenotype of microglia includes glycolysis as the dominant pattern for obtaining energy. Iron in microglia activates nicotinamide adenine dinucleotide phosphate oxidase (NOX2), which stimulates the formation of free radicals. Heme oxygenase 1(HO-1) in microglia increases the formation of free radicals and reduces the formation of glutathione peroxidase 4(GSHPx4). The sensitivity of microglia to ferroptosis and which drugs can inhibit it are currently being studied [37].

Iron is part of heme, which can affect mitochondrial function and antioxidant response. Free heme can cause the formation of free radicals. Iron–sulfur clusters are essential for the functioning of the mitochondrial respiratory chain. The entry of heme into the cell activates HO-1, which subsequently causes oxidative damage. HO-1 is expressed by cells of the phagocytic system, which includes microglia. However, protective effects of HO-1 in neurodegenerative diseases have also been observed [43].

Glutathione is the most important defense mechanism against ferroptosis. Ferritin deficiency induces ferroptosis because the transporter for the uptake of the amino acid cysteine, from which glutathione is synthesized, is not expressed. Ferritin is stored in the cell in three forms, one of which is mitochondrial ferritin, which protects mitochondria from free radicals by controlling free iron. Increased iron release from ferritin induces the ferroptosis process. As inhibitors of ferroptosis, deferoxamine, vitamins C and E, ferrostatin 1, lipostatin 1, ebselen (has the function of the enzyme GSHPx), and idebenone (synthetic coenzyme Q), as well as rosiglitazone, troglitazone and pioglitazone are proposed. Glia cells express growth and differentiation factor 15 (GDF15) which is important in mitochondrial dysfunction in the process of ferroptosis of neurons. Heme oxygenase can also be found in mitochondria, where its dual proferroptotic and antiferroptotic function has been observed. We can also observe transferrin as a marker of ferroptosis due to its transport function [44].

Studies are investigating the effect of deferoprone therapy, which is an iron chelator, as it has been shown to mobilize iron from focal lesions in neurodegenerative diseases [45].

Natalizumab has been shown to slow progression in the secondary progressive form of the disease. Tyrosine kinase inhibitors that inhibit microglia activation have been studied. Increased iron accumulation has been observed in the nuclei of the deep gray matter. Studies with iron chelators, such as deferoxamine, are being conducted [7].

## 6. Magnetic Resonance Findings in Multiple Sclerosis

Magnetic resonance imaging techniques used for a diagnosis of multiple sclerosis include spin echo T2 sequence, spin echo T1 sequence with gadolinium, FLAIR, diffusion techniques, magnetization transfer, spectroscopy, and functional MRI [46].

Conventional MRI does not detect changes at the cellular level. The newer QUEnch-assiSTed (QUEST) technique can detect changes in oxidative stress, the complete redox status. In vivo imaging of the proton exchange rate with endogenous contrast is a method for detecting oxidative stress. This proton exchange is increased in both active and chronic smoldering lesions in multiple sclerosis. The disadvantage of this technique is the long imaging time. The contrast compound 4-hydroxy-TEMPO (TEMPOL) is actually nitric oxide and in states of oxidative stress crosses the blood–brain barrier [14].

MR spectroscopy is a non-invasive method that displays metabolites in specific regions of the brain. It uses a standard technique and does not require contrast. The problem is that low-molecular compounds such as GSH exist in low concentrations, so larger samples need to be examined. Some metabolites overlap in the images. The reference metabolite against which everything is looked at is water. GSH is one of the best markers of redox balance in the cell. A decrease in GSH has been observed in the white matter in multiple sclerosis. In a study using the chemical change imaging technique, it was observed that GSH is reduced in patients with multiple sclerosis, and that it is lower in the progressive form compared to the relapsing form. In the secondary progressive form of the disease, patients with clinical exacerbation had a greater decrease in GSH levels compared to those without exacerbation. There is also the possibility of imaging oxidative stress outside the lesions themselves. The disadvantage of this technique is the need for a polarizer and multi-core scanner, which are not widely available [14,47,48].

Brain atrophy on magnetic resonance imaging is nonspecific and occurs in the last stage of the disease. Biomarkers are being sought in the early phase of the disease. Conventional magnetic resonance imaging is applicable in the relapsing form of multiple sclerosis. The finding is biologically nonspecific and time-dependent. It is used to assess response to therapy, prognosis and disability. It often fails to capture a snapshot of chronic neuroinflammation. Regional and global atrophy should be registered early in the course of the disease [14].

On magnetic resonance imaging, the most noticeable lesions can be found in the corpus callosum, which is the most important white matter because it contains pathways with myelinated axons [49]. In a study investigating the effect of multiple sclerosis on the size and morphology of the corpus callosum, the thickness of the corpus callosum was examined in different parts (the knee, body, and splenium). The results showed a significant reduction in the thickness of all parts of the corpus callosum in patients with multiple sclerosis compared with healthy controls. For the first time, the results showed that the reduction in the total area of the corpus callosum in patients with multiple sclerosis is not specific to a specific subregion of the corpus callosum, but it may be the result of the participation of all subregions of the corpus callosum, despite the difference in the degree of atrophy between the different subregions [50]. The corpus callosum is a biomarker and predictor of neurodegeneration. The problem is how to measure corpus callosum atrophy; so far, it has been measured manually through images, but automatic segmentation methods are being developed [51,52].

In a similar study, it was observed that the shape of the corpus callosum varies with physical and cognitive disability. In more severe disability, the corpus callosum is thinner, more atrophic, and has increased curvature. Cognitive disability is significantly correlated with the thinning of the corpus callosum. The shape of the corpus callosum differs in healthy and diseased individuals. The consequence of the thinning of the corpus callosum is ventriculomegaly [53]. The frontal and parietal lobes contain the majority of lesions in relapsing-remitting and secondary-progressive patients. The anterior parts of the corpus callosum showed the least volume change. The strongest predictors of atrophy of the middle and posterior parts of the corpus callosum were deep temporal lesions, occipital white matter lesions, and cortical lesions. Patients with secondary-progressive multiple sclerosis had lesions of significantly larger volume than patients with relapsing-remitting multiple sclerosis, except in the temporal and occipital cortex. In patients with secondary-progressive multiple sclerosis, the corpus callosum was significantly smaller than in patients with relapsing-remitting multiple sclerosis [54].

Brain atrophy in the caudate nucleus, putamen, thalamus, and hippocampus regions is the earliest predictor of disease progression and can be observed even in a clinically isolated syndrome. Thalamic atrophy occurs early in the course of the disease and is one of the strongest predictors of progression. Spinal cord atrophy is an important predictor of conversion to a secondary-progressive form of the disease, even in the absence of relapses and other disease activity. In multiple sclerosis, demyelination in the visual system involves the optic nerve, optic radiation, and lateral geniculate nucleus of the thalamus [55].

There is a paradox between MRI findings and the progression of disability. The presence of relapses and contrast-enhancing T1 or new or enlarged T2 lesions does not predict outcome or how disability will develop. Early relapses indicate faster progression, while late relapses do not affect the development of disability. Even patients with more than three relapses per year may have a mild course of the disease. Thus, the occurrence of relapses and MRI activity cannot predict outcomes in untreated patients, indicating that therapy does not affect the basic process of multiple sclerosis. Teriflunomide has been shown in studies to be effective in reducing brain volume loss.

The appearance of slowly growing lesions with a ring of microglia and active microglia in normal-appearing tissue are new features that are being recorded using advanced techniques such as magnetization transfer, diffusion tensor imaging, and T1 and T2 relaxometry. These slowly growing lesions have been observed early in both relapsing and progressive forms, suggesting that they may be a guide to the progression of multiple sclerosis. Changes in brain volume may be predictive [7].

A study was conducted at a university hospital in the Czech Republic where magnetic resonance imaging (MPRAGE—Magnetization Prepared Rapid Gradient Echo, GRE—gradient echo and FLAIR—Fluid Attenuated Inversion Recovery sequences) and an analysis of markers in the cerebrospinal fluid using antibodies (the ELISA method) were performed. The results obtained in patients with multiple sclerosis were reduced volumes of the thalamus, pulvinar and putamen and greater susceptibility of the caudate nucleus and globus pallidus internus. Here, it is suggested that iron deposition in these nuclei causes the process of ferroptosis and the creation of oxidative stress [19].

The neurodegeneration that occurs in the gray matter is irreversible, occurs early in the course of multiple sclerosis, and results in permanent disability [33].

Quantitative susceptibility mapping detects lesions surrounded by a ring of iron-laden microglia that show greater expansion over time compared to lesions without this ring. These lesions persist for years and often convert to T1 black holes [9].

Magnetic resonance imaging has shown iron accumulation in deep gray matter, while iron accumulation is reduced in normal-appearing white matter [13].

## 7. Conclusions and Future Perspectives

The important aspect of MS research is directed to remyelination process. Opicinumab, a humanized monoclonal antibody, was tested in clinical trials in context of remyelination. It targets leucine-rich repeat neuronal protein 1 (LINGO1) expressed on both neurons and oligodendrocytes and exerts inhibitory effects on myelin formation [56]. In a recent study, the muscarinic M1 receptor was investigated as a target with the aim of promoting remyelination. PIPE-307, a small molecule antagonist that crosses the blood–brain barrier, has been developed. In animal models, PIPE-307 has shown significant efficacy [57]. To stimulate myelination, B vitamins are recommended (the most important is pantothenic acid—vitamin B5), vitamins A, D, K, essential fatty acids, and protein intake, since methionine is an amino acid from which myelin is formed. Thyroid hormones stimulate remyelination through stimulation of oligodendrocyte progenitors. A common comorbidity is autoimmune thyroiditis with hypothyroidism. Appropriate thyroid hormone supplementation should be considered. The goal of therapy should also be to treat mitochondrial disfunction with a target in the NLRP3 inflammasome receptor structure [58]. Animal experiments have been conducted where mitochondrial transplantation was attempted to restore ATP production. The first evidence was obtained in mice with autoimmune experimental encephalomyelitis. It can be considered a potential form of therapy for patients with multiple sclerosis [59]. Hydroxychloroquine is being tested in a model of experimental autoimmune encephalitis where it has been shown to inhibit microglial activity, alleviate iron toxicity, and reduce disability. Statins are a group of drugs that penetrate the CNS and act by inhibiting microglial activation, cytokine release, proteases, and free radicals. They have been shown to reduce brain atrophy at an annual rate in the secondary-progressive form. Ibudilast is a phosphodiesterase inhibitor that antagonizes microglial activity and reduces neuronal death in tissue culture. Clemastine is a muscarinic antagonist that promotes oligodendrocyte differentiation and potentially leads to remyelination [60,61]. It should be studied whether the integration of the virus into the human genome has an impact on the process of demyelination and remyelination [62]. Monitoring metabolic pathways via magnetic resonance spectroscopy using carbon isotopes gives us useful information about processes in mitochondria [63]. The latest attempts at therapy involve the transplantation of neuronal precursors in animal models. The use of neural progenitors has also been attempted in human studies and has proven successful in reducing the rate of brain atrophy and increasing protective molecules in the cerebrospinal fluid of patients with multiple sclerosis [64,65,66]. The beneficial effect of melatonin, which is manifested in raising the levels of antioxidant enzymes catalase and superoxide dismutase and glutathione, has been studied, and its use in therapy should be considered [67,68,69].

The importance of neurofilament light chain (Nfl) and glial fibrillary acidic protein (GFAP) has been elucidated in a recent study, where higher levels of these biomarkers indicate a higher risk of disability progression [70]. The new diagnostic criteria, which are still under revision, will include Nfl, as well as oligoclonal bands [71]. The revision of the diagnostic criteria will emphasize the importance of diagnosing a radiologically isolated syndrome in the early stages of the disease [72]. Cortical lesions, gray matter volume changes, brain atrophy, corpus callosum index, thalamic volume changes, and spinal cord lesions are considered as prognostic factors [73]. Giovannoni et al. have developed a new concept of smoldering exacerbation associated with pseudorelapses. Pseudorelapse usually occurs when patients attempt to do something physically or mentally strenuous that they have not done for some time [74].

The development of therapeutic procedures should target smoldering lesions and microglia, the main figure mediating the generation of oxidative stress in the lesion and its surroundings. The ability of microglia to mobilize oligodendrocyte precursors should be investigated. In antioxidant defense, the replacement of antioxidant enzymes should be considered. One of the unresolved questions is how to protect demyelinated axons that are more susceptible to the influence of negative environmental effects. Due to the high involvement of glycolytic pathway activity in immune cells, glial cells, and neurons of patients with multiple sclerosis, inhibition of glycolysis and lactate formation should be seriously considered to further prevent the worsening of the state of oxidative stress.

In future studies, the corpus callosum should be monitored, as the most prominent white matter, because lesions in it reflect the functioning of the motor, sensory, auditory and visual systems, but also as a biomarker of neurodegeneration because it serves to monitor the course of the disease and has prognostic significance. The question arises as to whether stimulating remyelination would lead to a reduction in lesions in the corpus callosum and therefore atrophy.

There is also a need for neuroprotective therapy and stimulation of the phenomenon of neuroplasticity [7].

## Figures and Tables

**Table 1:** Biomarkers of oxidative stress in multiple sclerosis found in the articles.

Name of the Article	Biomarkers Found in the Article
Immunolgy and Oxidative Stress in Multiple Sclerosis: Clinical and Basic Approach, Ortiz G, 2013 [3]	NT and HNE ↑ in astrocytes and macrophages, ↑ plasma levels of cholesteryl ester hydroperoxide, oxidized LDL and other lipids ↑, pentane, ethane, MDA, and isoprostane ↑ in cerebrospinal fluid, NT ↑ in active lesions, SOD, CAT, NADH-2s and HO ↑ in the lesion, in astrocytes and macrophages, paraoxonase↓ (protective enzyme that hydrolyzes oxidized lipids) in the plasma, ↑ UA in lesions, GSH and α-tocopherol, ↑ activity of GR and GSHPx, ↑ UA in the cerebrospinal fluid, xanthine and hypoxanthine ↑ In the relapsing-remitting form, ↑ NO_3_, NO_2_, MDA and 4-HNE in the serum. iNOS ↑ in the lesions and CSF
Oxidative stress in multiple sclerosis-Emerging imaging techniques, Hollen C, 2022 [14]	↑ MDA in blood and cerebrospinal fluid, ↓ albumin in blood, ↑ 8-iso-PGF 2α in urine and cerebrospinal fluid, GSH 10-fold ↑ in the cells, ↓ GSH in the cerebrospinal fluid
Oxidative stress in multiple sclerosis: Central and peripheral mode of action, Ohl K, 2016 [12]	In the urine 8-isoPGF2α ↑, in the blood ↓ ferroxidase activity, ↓ antioxidant capacity, ↑ AOPP, ↓ SH, ↑ co Q10 and anti-oxLDL antibodies
Oxidative stress-related risk of the multiple sclerosis development, Vasic M, 2023 [15]	Measured: TAS, TOS, OSI, 8-oxo-dG and its relationship with creatinine, relationship with cigarette smoking Women ↑ risk associated with smoking, ↑oxidative markers, antioxidants ↓ compared to men, smokers ↑ oxidative markers
Evaluation of Selected Oxidant/Antioxidant Parameters in Patients with Relapsing-Remitting Multiple Sclerosis Undergoing Diesase-Modifying Therapies, Bizon A, 2022 [16]	Measured: SOD, GSHPx, CAT, IL-6, lipid peroxidation parameters, TAS, TOS IL-6, NO_2_, CAT ↑, and GSHPx (an enzyme that, together with catalase, converts hydrogen peroxide into water) ↓ in the relapsing-remitting form. In men, TAS ↑ compared to women, and CAT activity ↓
Mitochondrial dysfunction, oxidative stress, neuroinflammation, and metabolic alterations in the progression of Alzheimer’s disease: A meta-analysis of in vivo magnetic resonance spectroscopy studies, Song T, 2022 [17]	Myoinositol is a marker of glial cells and is actually part of the lipid layer membrane, participating in the formation of phosphatidyl inositol, which is a secondary messenger. ↑ myoinositol in both multiple sclerosis and Alzheimer’s disease
Exploring the Relationship between Antioxidant Enzimes. Oxidative Stress Markers, and Clinical profile in Relapsing -Remitting Multiple Sclerosis, Bizon A, 2023 [18]	Measured: AOPP and FRAP in patients with relapsing-remitting multiple sclerosis. AOPP ↑, and more so in men. There is a connection between AOPP and leukocyte count and CRP. The highest AOPP value was in those treated with GA and IFN β, while the lowest value was in those treated with DMF. There is a hint of differences due to sex hormones
Oxidative Stress Markers in Cerebrospinal Fluid of Newly Diagnosed Multiple Sclerosis Patients and Their Link to Iron Deposition and Atrophy, Burgetova A, 2022 [19]	In the cerebrospinal fluid, the following were measured: 8-OHdG,8-iso-PG, NGAL, PRDX-2, MDA, HAE. ↑ of 8-OHdG, PRDX2, MDA and HAE. 8-isoPG is significantly higher in the secondary progressive form of the disease, while initially this increase is smaller; here, it correlates negatively with the susceptibility of the globus pallidus externus. NGAL ↑ in progressive forms, NA ↓ it
Oxidative Stress marker Aberrrations in Multiple Sclerosis: A Meta-Analysis Study, Zhang S.-Y, 2020 [20]	Measured: SOD, MDA, GSH, TAS, TOS, CRP, Tg, albumin, AOPP, CAT, hydroxyguanosine, UA, ceruloplasmin, Tf, LDL, and chol. MDA and lipid hydroperoxide ↑, albumin ↓. Only ↑ MDA in the cerebrospinal fluid
Mitochondrial and metabolic disfunction of peripheral immune cells in multiple sclerosis, Wang P.-F, 2024 [21]	DMF ↓ intracellular GSH, ↑ free radical levels and ↓ oxygen utilization. In the CSF, memory B cells produce ↑ chol
Blood biomarkers fro assessment of mitochondrial dysfunction: An expert review, Hubens W, 2022 [22]	Biomarkers suggested: Pcr and AC
Mitochondrial dysfunction in neurodegenerative disorders, Klemmensen M.M, 2024 [23]	Measured: serum Fr ↑, LF ↑
Serum malondialdehyde as a lipid peroxidation marker in multiple sclerosis patients and its relation to disease characteristics, Nesma A.M. Ghonimi, 2021 [24]	MDA ↑ during relapse. In patients taking IFN β, this parameter is ↓ and correlates significantly with expanded disability status scale (EDSS)

Abbreviations: ↑—increased, ↓—decreased, NT—Nitrotyrosine, HNE—acrolein 4-hydroxy-2-nonenal, MDA—malonaldehyde, SOD—superoxide dismutase, CAT—catalase, NADH-2s—NADP quinone oxidoreductase and HO- heme oxygenase, UA—uric acid, GSH—glutathione, GR—glutathione reductase, GSHPx—glutathione peroxidase, NO_3_—nitrate, NO_2_—nitrite, iNOS— inducible nitric oxide synthase, 8-iso PGF 2α—8-iso-prostaglandin F2α, AOPP—protein oxidation product, SH—thyol groups, coQ10—coezyme Q10, anti-oxLDL antibodies—antioxidazed low-density lipoprotein antibodies, TAS—total antioxidant status, TOS—total oxidative status, OSI—oxidative stress index, 8-oxo-dG—8-oxo-7,8-dihydro-2′-deoxyguanosinecreatinine, IL-6—interleukin 6, FRAP—ferric-reducing antioxidant capacity, CRP—C reactive protein, GA—glatiramer acetate, IFN β—interferon β, DMF—dimethyl fumarate, 8-OH-dG—8-hydroxy-2′-deoxyguanosine, 8-iso-PG—8-iso prostaglandin F2α, NGAL—neutrophil lipocalin associated with gelatinase, PRDX2—peroxiredoxin-2, NA—natalizumab, Tg—triglyceride, LDL—low-density lipoprotein, Tf—transferrin chol—cholesterol, Pcr—phosphocreatine, AC—acylcarnitine, Fr—ferritin, LF—lipofuscin, CSF—cerebrospinal fluid.

## Data Availability

Data sharing not applicable to this article as no datasets were generated or analyzed during the current study. Data available on request.
